# Retrospective and prospective evaluation of the Carbapenem inactivation method for the detection of carbapenemase-producing *Enterobacteriaceae*

**DOI:** 10.1371/journal.pone.0170769

**Published:** 2017-02-03

**Authors:** Lauraine Gauthier, Remy A. Bonnin, Laurent Dortet, Thierry Naas

**Affiliations:** Bactériologie-Hygiene unit, APHP, Bicêtre Hospital, EA7361 “Structure, dynamic, function and expression of broad spectrum β-lactamases”, Paris-Sud University, LabEx Lermit, Faculty of Medecine, French National Reference Center for Antibiotic Resistance: Carbapenemase producing Enterobacteriaceae, Le Kremlin-Bicêtre, France; Ross University School of Veterinary Medicine, SAINT KITTS AND NEVIS

## Abstract

**Background:**

There is an urgent need for accurate and rapid diagnostic tests to identify carbapenemase producing enterobacteria (CPE). Here, we have evaluated the Carbapenem Inactivation Method (CIM) test to detect CPEs from cultured colonies.

**Methods:**

A total of 256 enterobacterial isolates were used to evaluate the performance of the CIM in comparison to Carba NP test and molecular detection used a reference method. Ninety three well-characterized isolates (including 29 non-CPE and 63 CPEs of worldwide origin) with decreased susceptibility to at least one carbapenem were used to (i) evaluate the efficacy of CIM test and (ii) to compare it to the Carba NP test. We also tested different carbapenems to determine the best substrate for this test. Finally, the CIM test was then evaluated prospectively against 164 isolates referred to the French National Reference Center (NRC) for Antimicrobial Resistance from may 2016 to july 2016.

**Results:**

Based on the results of this retrospective study, sensitivity and specificity of the CIM and the Carba NP test were 92.1% and 100%, respectively. We demonstrated that the meropenem was the best substrate to perform the CIM test since sensitivity and specificity were 81.1% and 100% using ertapenem disk, and 100% and 65,6% using imipenem disk, and respectively. Taking in account the results of retrospective and prospective studies, CIM and Carba NP tests have similar sensitivity, specificity, positive predictive value and negative predictive values being 96.3%, 98.9%, 99.0% and 98.4% for the CIM test versus 96.9%, 100%, 100% and 100% for the Carba NP test.

**Conclusions:**

Our results confirm that the CIM test may be a useful tool for the reliable confirmation of carbapenemase-activity in enterobacterial isolates, especially in clinical microbiological laboratories with limited resources, no trained personnel, and no specialized equipment.

## Introduction

Carbapenemase producers are increasingly reported worldwide in *Enterobacteriaceae* [1]. They are most often resistant to many classes of antimicrobial molecules, and thus represent a major public health concern. Their identification is of primary importance for the choice of appropriate therapeutic schemes and the implementation of proper infection control measures [1,2]. Identification based solely on antibiotic susceptibility testing is not easy and a recently described algorithm, showed that at least 66% of *Enterobacteriaceae* with a reduced susceptibility at least one carbapenem required confirmatory testing [3]. Thus, reliable and simple confirmatory tests are required.

Many phenotypic or advanced molecular-based methods have been proposed for the detection of carbapenemase production. The phenotypic confirmation tests for detecting carbapenemases include inhibition tests of carbapenemase activity [4], detection of carbapenem hydrolysis using MALDI-TOF MS [5], biochemical tests (e.g. Carba NP Test and derivatives) [6–8] or electrochemical measurement (BYG test; [9]). These tests are able to detect the presence of a carbapenemase activity and sometimes to discriminate between Ambler class A and Ambler class B carbapenemases (e.g inhibition tests and Carba NP test II; [10]). A new immunochromatographic assay, called OXA-48 *K*-SeT and OXA-48/KPC *K*-SeT, aiming to detect OXA-48-like carbapenemase producing enterobacteria (CPE) or OXA-48 and KPC variants, respectively from solid cultures has also been shown to be very useful especially in settings with high OXA-48 prevalence [11,12]. However, the gold standard remains molecular methods for the detection of CPE-producers [2,13–15].

The recently described Carbapenem Inactivation Method (CIM) has been developed as a phenotypic technique for detecting carbapenemase activity from bacterial culture [16]. It was reported to be 100% sensitive and specific for CPE detection. In addition, it does not require trained personnel or specific equipment, and is cost-effective. We compared the performances of the CIM test for detection of CPE with those of the Carba NP test used as reference (systematically tested on isolates referred to the NRC) against various *Enterobacteriaceae*, including combinations of bacterial/resistance mechanisms that were not previously evaluated.

## Materials and methods

### Bacterial isolates

A total of 256 enterobacterial isolates were used to evaluate the performance of the CIM test [16] in comparison with a home-made technique of the Carba NP test [6]. 92 strains had previously been characterized for their β-lactamase content at the molecular level. This collection included 29 non-carbapenemase producers with decreased susceptibility to at least one carbapenem molecule (imipenem, meropenem, or ertapenem) and 63 were CPEs originating from our strain collection (Tables 1 and 2). These CPEs included 25 OXA-48 producers, 5 KPC producers, 12 NDM producers, 7 VIM producers, 5 IMP producers, 3 IMI producers, 2 SME producers, 1 GES-5 producer, 1 GIM-1 producer, 1 FRI-1 producer and 1 NMC-A producer. The CIM test was then evaluated prospectively against 164 Carbapenem Resistant Enterobacterial isolates (CREs) (decreased susceptibility to at least one carbapenem molecule) referred to the French National Reference Center for Antimicrobial Resistance from 15 March 2016 to 15 April 2016 (Table 3).

**Table 1:** Carbapenemase-producing *Enterobacteriaceae* subjected to the CIM method, compared to the Carba NP test.

Strain no.	Species	Carbapenemase	other β-lactamase genes	MICs (mg/L)*			CIM (inhibitory diameter, mm)			CIM decision	Carba NP test
				IMP	ERT	MER	ERT	IMP	MER		
**Class A carbapenemases**											
1I3	*E*. *cloacae*	IMI-1		>32	8	4	6	6	6	+	+
1I4	*E*. *asburiae*	IMI-2		>32	>32	>32	6	6	6	+	+
28D8	*E*. *cloacae*	IMI-3		>32	>32	>32	6	6	6	+	+
1G1	*K*. *pneumoniae*	KPC-2	SHV-11, TEM-1	4	6	8	6	6	6	+	+
1F5	*K*. *pneumoniae*	KPC-2	SHV-11, TEM-1, CTX-M-2	16	24	32	6	6	6	+	+
1F3	*E*. *coli*	KPC-2	TEM-1 + OXA-9	2	1.5	1	6	6	6	+	+
1F9	*K*. *pneumoniae*	KPC-2	SHV-11 + TEM-1 + SHV-12 + OXA-9	4	24	2	6	6	6	+	+
1G2	*K*. *pneumoniae*	KPC-3	TEM-1 + SHV-1 + CTX-M-15 + OXA-9	4	>32	8	6	6	6	+	+
3B7	*K*. *pneumoniae*	GES-5	SHV-12	0.38	1	0.19	6	6	6	+	+
1I6	*E*. *cloacae*	NMC-A		>32	1.5	0.75	6	6	6	+	+
1I7	*S*. *marcescens*	SME-1		>32	8	8	6	6	6	+	+
1I8	*S*. *marcescens*	SME-2		>32	8	8	6	6	6	+	+
1I10	*E*. *cloacae*	FRI-1		>32	>32	16	6	6	6	+	+
**Class B carbapenemases**											
1A1	*E*. *coli*	NDM-1	OXA-1 + OXA-10 + CMY-16 + TEM-1	1	3	1	6	6	6	+	+
1A5	*E*. *coli*	NDM-1	CTX-M-15 + TEM-1	16	>32	16	6	6	6	+	+
1B1	*K*. *pneumoniae*	NDM-1	CTX-M-15 + SHV-11 + OXA-1	2	8	3	6	6	6	+	+
1B4	*K*. *pneumoniae*	NDM-1	OXA-1 + SHV-11	1.5	6	2	6	6	6	+	+
1C1	*Salmonella*	NDM-1	CTX-M-15 + TEM-1 + OXA-1 + OXA-9 + OXA-10	4	6	3	6	6	6	+	+
1B5	*K*. *pneumoniae*	NDM-1	OXA-1 + CTX-M-15 + TEM-1 + SHV-28 + OXA-9 + CMY-6	1	8	4	23	6	26	-	+
1B9	*P*. *stuartii*	NDM-1	OXA-1 + CMY-6 + TEM-1	12	0.38	1.5	28	6	25	-	+
1B10	*P*. *rettgeri*	NDM-1	CTX-M-15	3	0.5	1.5	27	6	23	-	+
1A6	*E*. *coli*	NDM-4	CTX-M-15 + OXA-1	>32	>32	>32	6	6	6	+	+
1A8	*E*. *coli*	NDM-5	TEM-1 + CTX-M-15	>32	>32	>32	6	6	6	+	+
1A9	*E*. *coli*	NDM-6	CTX-M-15 + OXA-1	6	32	8	6	6	6	+	+
3A5	*E*. *coli*	NDM-9	OXA-1, TEM-1B	6	4	4	6	6	6	+	+
1C7	*K*. *pneumoniae*	VIM-1	SHV-12	>32	>32	>32	6	6	6	+	+
1D5	*E*. *cloacae*	VIM-1	SHV-70	1	0.38	0.5	24	6	6	+	+
1C3	*E*. *coli*	VIM-1	CMY-13	3	1.5	1	25	6	6	+	+
3D3	*P*. *mirabilis*	VIM-1	CTX-M-15, TEM-2	3	0.12	0.19	27	6	22	-	+
3B8	*E*. *cloacae*	VIM-2	TEM-1 + OXA-9 + ACT-15	0.5	0.75	0.19	27	6	22	-	-
1D6	*E*. *cloacae*	VIM-4	CTX-M-15 + TEM-1 + SHV-31	3	2	1	6	6	6	+	+
1D4	*K*. *pneumoniae*	VIM-19	CTX-M-3 + TEM-1 + SHV-1	8	16	4	6	6	6	+	+
1 E2	*K*. *pneumoniae*	IMP-1	TEM-15	8	3	2	6	6	6	+	+
1 E3	*K*. *pneumoniae*	IMP-1	TEM-15 + CTX-M-15	1.5	4	2	6	6	6	+	+
3D4	*K*. *pneumoniae*	IMP-4		0.25	0.5	0.38	6	6	6	+	+
1 E8	*E*. *cloacae*	IMP-8	SHV-12	0.75	0.5	0.5	6	6	6	+	+
1 E9	*S*. *marcescens*	IMP-11		8	>32	2	6	6	6	+	+
1 E10	*E*. *cloacae*	GIM-1		2	>32	6	6	6	6	+	+
**Class D carbapenemases**											
2A1	*E*. *coli*	OXA-48	CTX-M-15	3	16	1	18	6	6	+	+
2A4	*E*. *coli*	OXA-48	CTX-M-24 + TEM-1	0.25	0.5	0.19	6	6	6	+	+
2A6	*E*. *coli*	OXA-48	CTX-M-15	0.5	1	0.25	6	6	6	+	+
2A7	*K*. *pneumoniae*	OXA-48		0.5	2	0.5	6	6	6	+	+
2A8	*K*. *pneumoniae*	OXA-48	TEM-1	0.38	1	0.5	6	6	6	+	+
2A9	*K*. *pneumoniae*	OXA-48	CTX-M-15	2	3	2	6	6	6	+	+
2B3	*K*. *pneumoniae*	OXA-48	SHV-11	0.5	0.75	0.25	6	6	6	+	+
2B6	*E*. *cloacae*	OXA-48	TEM-1 + CTX-M-15 + OXA-1	0.5	2	0.5	6	6	6	+	+
2B10	*C*. *koseri*	OXA-48	TEM-1	0.75	2	0.38	6	6	6	+	+
2C1	*C*.*freundii*	OXA-48	SHV-12 + TEM-1	0.75	1.5	0.38	6	6	6	+	+
3C7	*P*. *mirabilis*	OXA-48		0.75	0.06	0.12	6	6	6	+	+
2C5	*K*. *pneumoniae*	OXA-181	SHV-11 + TEM-1 + CTX-M-15 + NDM-1 + OXA-1	3	>32	4	6	6	6	+	+
2C9	*K*. *pneumoniae*	OXA-181	SHV-11 + CTXM-15 + OXA-1	0.5	2	0.5	27	6	6	+	+
2D1	*C*.*freundii*	OXA-181	NDM-1 + OXA-1 + OXA-9 + OXA-10 + CTX-M-15 + TEM-1	16	>32	16	6	6	6	+	+
2C4	*E*. *coli*	OXA-181		0.5	1.5	0.25	19	6	6	+	+
3C9	*K*. *pneumoniae*	OXA-181	CTX-M- 15, OXA-1	0.38	0.38	0.12	6	6	6	+	-
2D2	*K*. *pneumoniae*	OXA-204	CMY-4	0.5	2	0.5	6	6	6	+	+
2D4	*E*. *coli*	OXA-204	CMY-4+ CTX-M-15 + OXA-1	0.5	2	0.25	6	6	6	+	+
2J6	*C*.*freundii*	OXA-372	CMY-135+OXA-10+MOX-9	3	2	0.5	6	6	6	+	+
2C2	*K*. *pneumoniae*	OXA-162	TEM-1 + SHV-11	4	8	1	6	6	6	+	+
2D9	*E*. *coli*	OXA-244	TEM-1 + CMY-2	0.5	2	0.5	21	6	6	+	+
2D10	*E*. *coli*	OXA-244	TEM-1 + CMY-2	0.5	1.5	0.5	6	6	6	+	-
3C10	*E*. *coli*	OXA-244		0.19	1.5	0.25	21	6	6	+	+
3D1	*E*. *coli*	OXA-244		0.19	1.5	0.25	6	6	6	+	-
3D2	*E*. *coli*	OXA-244		0.25	1.5	0.25	6	6	6	+	-

* MIC, minimal inhibitory concentration, IMP, imipenem, MER, meropenem, ERT, ertapenem. False negative results for the CIM and/or the Carba NP test are highlighted.

**Table 2:** Non–carbapenemase-producing *enterobacteriaceae* subjected to the CIM method, compared to the CarbaNP test.

Strain no.	Species	β-lactamases	MICs (mg/L)*			CIM(inhibitory diameter, mm)			CIM decision	Carba NP test
			IMP	ERT	MER	ERT	IMP	MER		
**ESBL + decreased membrane permeability**										
2I3	*K*. *pneumoniae*	CTX-M-15 + TEM-1 + SHV-11	3	>32	6	31	23	29	-	-
2H9	*K*. *pneumoniae*	CTX-M-15 + TEM-1 + SHV-1	0.25	1	1	32	25	28	-	-
3D6	*K*. *pneumoniae*	CTX-M-15, TEM-1B, SHV-28	1	>32	4	29	22	27	-	-
2G4	*K*. *pneumoniae*	SHV-76	0,125	0,75	0,25	32	23	30	-	-
3C3	*K*. *pneumoniae*	CTX-M-1 + LEN-16	1,5	>32	6	33	20	29	-	-
3C4	*K*. *pneumoniae*	CTX-M-15 + TEM-1B + SHV-11	0,5	12	3	32	22	29	-	-
3D6	*K*. *pneumoniae*	CTX-M-15 + TEM-1B + SHV-28	1	>32	4	29	22	27	-	-
3D7	*K*. *pneumoniae*	CTX-M-15 + TEM-1B + SHV-83	4	>32	>32	32	25	29	-	-
3D8	*C*. *freundii*	CTX-M-15 + TEM-1b + CMY-48	>32	>32	>32	34	23	28	-	-
3C5	*E*. *cloacae*	CTX-M-15 + TEM-1B	0.19	0.5	0.06	34	23	33	-	-
**Plasmid-mediated AmpC or chromosomal AmpC + decreased membrane permeability**										
2G4	*E*. *cloacae*	Overexpressed cephalosporinase	0.19	1.5	0.12	30	6	29	-	-
2G5	*E*. *cloacae*	Overexpressed cephalosporinase	0.5	4	0.75	31	6	29	-	-
2G6	*E*. *cloacae*	Overexpressed cephalosporinase	1.5	0.75	0.25	31	18	28	-	-
3C1	*E*. *aerogenes*	Overexpressed cephalosporinase	4	>32	3	30	6	27	-	-
3B10	*K*. *pneumoniae*	CMY-16 + SHV-11 + TEM-1B	0,19	6	1	31	23	29	-	-
3D5	*K*. *pneumoniae*	SHV-1, DHA-1	>32	>32	>32	33	6	30	-	-
3E1	*E*. *coli*	CMY-2	>32	>32	8	31	13	28	-	-
**ESBL + plasmid-mediated AmpC or chromosomal AmpC + decreased membrane permeability**										
2I9	*E*. *cloacae*	Overexpressed cephalosporinase + CTX-M-15	1.5	6	1	31	22	30	-	-
2I10	*E*. *cloacae*	Overexpressed cephalosporinase + CTX-M-15	2	8	1	33	20	29	-	-
2J1	*E*. *cloacae*	Overexpressed cephalosporinase + CTX-M-15	3	12	2	33	22	29	-	-
2J2	*C*. *freundii*	Overexpressed cephalosporinase + CTX-M-15 +TEM-3	1	8	1	30	6	27	-	-
**Extended spectrum oxacillinases**										
2J3	*K*. *pneumoniae*	OXA-163	0.5	0.38	0.12	31	18	29	-	-
2J4	*E*. *cloacae*	OXA-163	0.5	2	0.19	31	6	28	-	-
2J5	*S*. *marcescens*	OXA-405	0.5	0.75	0.19	31	6	25	-	-
**Other mechanisms + decreased membrane permeability**										
3D9	*E*. *coli*	OXA-1	0,38	4	0,5	30	23	27	-	-
3D10	*K*. *oxytoca*	Overexpressed OXY-2-3	1	4	4	30	6	26	-	-
3E2	*E*. *cloacae*	OXA-35 + ACT-6	24	>32	>32	32	6	26	-	-
3E3	*E*. *coli*	TEM-1B	2	16	3	34	20	28	-	-
2J10	*K*. *pneumoniae*	SHV-1	1	12	2	33	6	30	-	-

* MIC, minimal inhibitory concentration, IMP, imipenem, MER, meropenem, ERT, ertapenem.

**Table 3:** *Enterobacteriaceae* isolates prospectively subjected to the CIM method, compared to the CarbaNP test.

β-lactamase	Species	No.	CIM		Carba NP test
			Meropenem inhibitory diameter, (mm)	Decision	
**Carbapenemase—Class A**					
IMI-1	*E*. *cloacae*	3	6	+	+
IMI-2	*E*. *cloacae*	1	6	+	+
KPC-2	*K*. *pneumoniae*	3	6	+	+
	*E*. *coli*	1	6	+	+
KPC-3	*K*. *pneumoniae*	1	6	+	+
	*C*. *freundii*	1	6	+	+
GES-5	*K*. *pneumoniae*	2	6	+	+
**Carbapenemase—Class B**					
NDM-1	*K*. *pneumoniae*	7	6	+	+
	*K*. *pneumoniae*	1	26	-	**+**
	*M*. *morganii*	4	6	+	+
	*E*. *cloacae*	1	6	+	+
	*E*. *coli*	1	6	+	+
NDM-4	*K*. *pneumoniae*	1	6	+	+
NDM-5	*E*. *coli*	2	6	+	+
NDM-6	*K*. *pneumoniae*	1	6	+	+
NDM-7	*E*. *coli*	2	6	+	+
VIM-1	*E*. *cloacae*	3	6	+	+
	*K*. *pneumoniae*	1	6	+	+
VIM-19	*K*. *pneumoniae*	1	6	+	+
IMP-10	*S*. *marcescens*	2	6	+	+
**Carbapenemase—Class D**					
OXA-48	*K*. *pneumoniae*	20	6	+	+
	*K*. *oxytoca*	4	6	+	+
	*E*. *coli*	16	6	+	+
	*E*. *cloacae*	3	6	+	+
	*C*. *freundii*	3	6	+	+
	*C*. *koseri*	2	6	+	+
	*C*. *braakii*	1	6	+	+
	*S*. *marcescens*	3	6	+	+
OXA-181	*E*. *coli*	5	6	+	+
OXA-204	*E*. *coli*	1	6	+	+
OXA-232	*K*. *pneumoniae*	1	6	+	+
OXA-181 + NDM-5	*E*. *coli*	1	6	+	+
OXA-232 + NDM-1	*K*. *pneumoniae*	1	6	+	+
**Non carbapenemase producers**					
ESBL	*K*. *pneumoniae*	16	28 -> 30	-	-
	*K*. *oxytoca*	1	28	-	-
	*E*. *coli*	5	27 -> 30	-	-
	*E*. *cloacae*	2	29 -> 30	-	-
Overexpressed cephalosporinase	*E*. *cloacae*	19	23 -> 29	-	-
	*C*. *freundii*	1	6	+	-
	*E*. *coli*	1	26	-	-
	*H*. *alvei*	1	27	-	-
	*S*. *marcescens*	1	28	-	-
Overexpressed cephalosporinase + ESBL	*E*. *cloacae*	6	27 -> 31	-	-
	*C*. *freundii*	1	27	-	-
	*E*. *coli*	2	27 -> 28	-	-
other / unknown mechanism(s)	*K*. *pneumoniae*	4	27 -> 30	-	-
	*C*. *freundii*	1	29	-	-
	*M*. *morganii*	1	26	-	-
	*P*. *vulgaris*	1	28	-	-
	*S*. *marcescens*	1	26	-	-

False negative or positive results for the CIM and/or the Carba NP test are highlighted.

### Antimicrobial susceptibility testing

Antimicrobial susceptibility testing using disc diffusion method and minimal inhibitory concentrations (MICs) using the E-test technique (bioMérieux, Marcy L’Etoile, France) were determined on Mueller-Hinton (MH) agar (Bio-Rad, Marnes-La-Coquette, France), and interpreted according to EUCAST guidelines, as updated in 2016 (http://www.eucast.org). Clinical carbapenem breakpoints for susceptibility/resistance were ≤ 2/>8 mg/l for imipenem and meropenem and ≤0,5/>1 mg/l for ertapenem.

### Carbapenemase activity testing

The updated version of the Carba NP test was used and interpreted as previously described [6]. The test was performed on bacterial colonies recovered from UriSelect^®^ 4 medium (Biorad, Marne La Coquette, France). Reading was performed after 2 h as recommended [3].

The CIM was performed using meropenem disks (Oxoid, Dardilly, France) as decribed [16], with overnight solid cultures. Briefly, the CIM is based on *in vitro* inactivation of the meropenem contained in a 10μg charged disk by carbapenemase-producing strains. This disk is placed in 400 μl bacterial suspension for 2h and susequently placed on a plate inoculated with a bacterial suspension of *E*. *coli* ATCC 29522, a carbapenem-susceptible strain. If the tested isolate produces a carbapenemase, the susceptible strain may grow contact to the disk. On the opposite, if the tested bacteria had decreased susceptibility to carbapenems without carbapenemase activity, an inhibition zone around the disk can be evidenced. In order-to determine the best carbapenem to use in this assay, we have also tested imipenem and ertapenem disks using the same protocol on the 92 well-characterized isolates. When false-negative or false-positive results were obtained either with the CIM or the Carba NP test, the two phenotypic methods were repeated in triplicate. If discrepancy was still observed, false positive isolates underwent whole genome sequencing, in order-to rule out the possibility of a carbapenemase that is not detect using our in-house PCR approach.

### Molecular identification of the carbapenemase gene

The CIM and the Carba NP test results were compared with those obtained using an in-house PCR-sequencing that was used as the gold standard [17]. The targeted carbapenemase genes were *bla*_OXA-48-like_, *bla*_KPC-like,_ *bla*_NDM-like_, *bla*_IMP-like_ and *bla*_VIM-like_, *bla*_IMI-like_ and *bla*_GES-like._ Total DNA was extracted from colonies using the Ultraclean Microbial DNA Isolation Kit (MO BIO Laboratories, Ozyme, Saint-Quentin, France) following the manufacturer’s instructions. Specific primers were used to detect the most common carbapenemase genes [17]. both strands of the PCR products were sequenced with an automated Applied Biosystems sequencer (ABI PRISM 3100, Applied Biosystems, Les Ulis, France). The nucleotide and the deduced protein sequences were analysed using software available on the β-lactamase database (BLDB; http://www.bldb.eu).

## Results

### Retrospective evaluation on the strain collection

The CIM test was first evaluated on 92 characterized strains (Tables 1 and 2). We have deliberately included strains producing OXA-48-like enzymes for which the Carba NP test gave a false negative result, as it is a common criticism [18–20], and some CPE with low carbapenemase activity. Based on the results of this retrospective study, sensitivity and specificity of the CIM and the Carba NP test were 92.1% [confidence interval 95% (CI95) = 82.7%-96.6%] and 100% [CI95 = 88.7%-100%], respectively.

All non-carbapenemase producers (n = 29) were negative using both phenotypic methods (Table 2), even though they had high MICs for carbapenems. The only discrepancy was a *K*. *oxytoca* with overexpressed OXY-2-3 initially CIM-positive, but the analysis was repeated and CIM was negative the second and the third time.

All class A (IMI-1/2, KPC-2/3, GES-5, NMC-A, SME-1/2 and FRI-1) and OXA-48-like carbapenemases were detected by the CIM test. Of note 5 OXA-48 like enzymes (3 OXA-244 and 2 OXA-181), which gave uncertain or false-negative results with the Carba NP test were positive for the CIM. On the other hand, the CIM did not detect 3 NDM-1-producing *Enterobacteriaceae* (1 *K*. *pneumoniae*, 1 *P*. *stuartii* and 1 *P*. *rettgeri*), which were repeatedly negative for the CIM, while the Carba NP test was positive. Similarly, a VIM-1-producing *P*. *mirabilis* isolate was also repeatedly negative for the CIM and positive for the Carba NP test. Finally, a VIM-2-producing *E*. *cloacae* isolate with very low carbapenemase activity, with MICs for Imipenem and Meropenem remaining in the susceptibility range, was negative by both methods.

### Evaluation of the best substrate for the CIM test

In order-to evaluate whether higher sensitivity and specificity may be obtained changing the carbapenem substrate, we have tested ertapenem and imipenem disks (Tables 1 and 2). Based on the results of this retrospective study, sensitivity and specificity were 81.1% [CI95 = 70.0%-88.8%] and 100% [CI95 = 88.7%-100%] using ertapenem disk, and 100% [CI95 = 94.3%-100%] and 65.6% [CI95 = 47.3%-80%] using imipenem disk, and respectively.

Although, the sensitivity reached 100%, an increase of the false-negative results was observed using the ertapenem disk compared to the meropenem. Additive false negative results were obtained for 4 VIM producers and 5 OXA-48-like producers.

**Fig 1:**
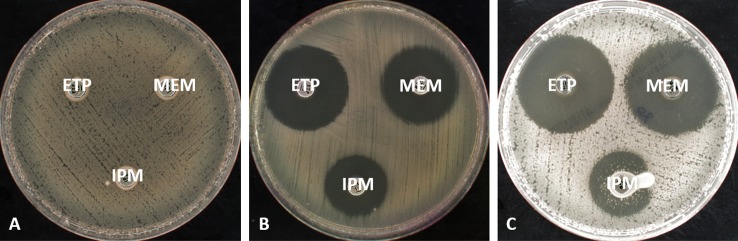
A: OXA-48 producing *E*. *coli*, CIM positive; B: CTX-M15 producing *K*. *pneumoniae*, CIM negative; C: Overexpressed cephalosporinase + CTX-M-15 producing *E*. *cloacae*, CIM negative, presence of microcolonies in the inhibitory diameter of imipenem disk. *ETP*: *Ertapenem; IPM*: *Imipenem; MEM*: *Meropenem*

Use of imipenem increased the sensitivity to 100% (vs 92.1% with the meropenem disk) but the specificity decreased to 65.6% (vs 100% with either meropenem or ertapenem disks). In addition, in some CIM-negative isolates, microcolonies were detected in the inhibitory diameter (5/19 isolates), making interpretation difficult (Fig 1C). False positive results were especially observed with AmpC over-producers (4/7), as already reported [16].

In the light of these results, meropenem disk and absence / presence of an inhibition zone remains the best interpretation criteria.

### Prospective analysis

Among the 164 consecutive *Enterobacteriaceae* isolates tested prospectively, 100 were CPE (59 OXA-48-like, 6 KPC, 20 NDM, 2 OXA-48-like + NDM, 5 VIM, 2 GES-5, 4 IMI and 2 IMP) while 64 were confirmed as non-carbapenemase producers using Carba NP test, and subsequent PCR, used as reference method (Table 3). One false negative and one false positive results were obtained with CIM, a NDM-1-producing *K*. *pneumoniae* and a *C*. *freundii* overexpressing its naturally-occurring cephalosporinase, respectively. On this prospective collection the Carba NP was 100% specific and 100% sensitive. Isolates with false positive results as compared to PCR results, were sequenced entirely. It allowed us to rule out the presence of a carbapenemase that was not detected using our PCR assay (data not shown).

According to this prospective study, CIM has a sensitivity value of 99.0% [CI95 = 94.6%-99.8%], a specificity value of 98.4% [CI95 = 91.7%-99.7%], a positive predictive value (PPV) value of 99.0% [CI95 = 94.6%-99.8%] and a nagative predictive value (NPV) value of 98.4% [CI95 = 91.7%-99.7%], compared to the Carba NP test which has 100% sensitivity, specificity, PPV and NPV.

Taking into account the results of the retrospective and the prospective study CIM test and Carba NP test have quite similar performances. Sensitivity was 96.3% [CI95 = 92.2%-98.3%] for the CIM test versus 96.9% [CI95 = 93.0%-98.7%] for the Carba NP test, and the specificity was 98.9% specificity [CI95 = 94.2%-99.8%] for the CIM test versus 100% [CI95 = 96.1%-100%] for the Carba NP test. Since NPV and PPV can be calculated only with the prospective results, the Carba NP test performed slightly better than the CIM test with 100% PPV and NPV compare to 99.0% and 98.4% for the CIM test.

## Discussion

Overall, our results indicate that the CIM has high sensitivity and specificity for CPE detection, as already reported [6,7]. It has significant advantages shared by the CarbaNP test being low cost and simplicity of implementation. In addition, it is very easy to interpret, the indicator *E*. *coli* strain grows directly contact to the meropenem disk (inhibitory diameter = 6 mm) with isolates having a carbapenemase activity, while a clear inhibition zone (most often > 20 mm) is obtained with non-carbapenemase producers. This ease of interpretation is a major advantage over the CarbaNP test, for which the colour change may be difficult to appreciate for non-experienced personal [18–20]. In addition, the CIM was positive for OXA-48 like producing isolates, which gave false-negative results with the Carba NP test. This is a particularly interesting point since these OXA-type carbapenemases may be phenotypically difficult to detect when no other β-lactamases are associated.

However, we did not find a 100% sensitivity as claimed by van der Zwaluw et al. [16]. Particularly, false-negative results were obtained with NDM-1 producing strains. Another study from Turkey also found a 100% sensitivity [21], but only one NDM-1-producing strain was included. The prevalence of NDM-1 is increasing in many European countries [22,23], and especially in France, where 15% of the CPE at the French NRC are NDM-like producers (in contrast to 4% in 2012) (T. Naas, personal comm.).

The main disadvantage of the CIM test resides in the need for two-hours incubation and a subsequent overnight culture. Although, it was reported that CIM was able to detect carbapenemase activity within eight hours [16], we found that the test was not interpretable within this time frame (data not shown). Accordingly, the CIM test is not a rapid detection assay of CPE, since results are not obtained the same day. In comparison, the CarbaNP test provides results within two hours, but in many cases (e.g. KPC, MBLs) positivity can be obtained after less than 15 minutes. Furthermore, the CIM test needs the handling of highly concentrated bacterial suspensions, and manipulation of contaminated antibiotic disks with iron tweezers. This implies thorough decontamination of the material to avoid cross-contamination.

In conclusion, we have confirmed that the CIM test might be a cheap and useful tool for the reliable confirmation of carbapenemase-producing *Enterobacteriaceae*, especially in clinical microbiological laboratories with limited resources, no trained personnel, and no specialized equipment to detect carbapenemase activity. Zwaluw et al also reported high performance of the CIM on non-fermenters (98,8% sensitivity), which has not been yet confirmed by other studies but might be of interest in the light of results on *Enterobacteriaceae*.

This test is really inexpensive since 5X50 meropenem disks cost c.a. $12 and only one is necessary to test a bacteria and one MH TSA plate, thus a single test cost about 0.048 $ + 0.43 $ thus c.a. 0.45 USD. Moreover, no additional reagents are required for this test.The in-house method of the CarbaNP test is cheap as well but requires more equipment/reagent (PH meter, Red Phenol, buffers, imipenem powder, etc..) and its cost was estimated about???. Most often, microbiological laboratories use the commercially available CarbaNP test test, which is more expensive (RAPIDEC (®) CARBA NP (bioMérieux) costs c.a. 7$ tests). The limitation of the CIM in term of clinical performance is the delay in obtaining the result after isolation of a bacterial colony, which is not compatible with rapid implementation of proper infection control measures, such as reinforced contact precautions, or cohorting of colonized/infected patients. Nevertheless, this test could be performed in parallel to a classical antibiogram on any bacteria growing on CPE screening selective media. Thus, the CIM test result would be available at the same time as the antibiogram. This test has the potential to be used a confirmatory test in many settings were novel, and expensive commercially detection tests are not available. Finally, molecular confirmatory tests are necessary to identify the carbapenemase-producing genes, and to validate the CIM-positive results.
